# Lyme Neuroborreliosis in the Context of Dementia Syndromes

**DOI:** 10.7759/cureus.67057

**Published:** 2024-08-17

**Authors:** Dzhaner H Bashchobanov, Eva Stamatova, Radina Andonova, Elena Dragusheva, Veronika Gadzhovska, Georgi Popov

**Affiliations:** 1 Medicine, Medical University of Sofia, Sofia, BGR; 2 Infectious Diseases, Sofiamed, Sofia, BGR

**Keywords:** microbial pathogenesis, nervous system lyme disease, alzheimer's disease, dementia syndromes, lyme neuroborreliosis, lyme disease

## Abstract

Lyme disease (LD) can affect the skin, joints, heart, and nervous system as a multisystemic condition. The cause of the illness is the spirochete of the genus *Borrelia*. These pathogens can affect the skin, joints, heart, and nervous system. Lyme neuroborreliosis (LNB) is the term for the disease, which occurs when the nervous system gets involved. Regarding geographical distribution, LNB is more prevalent in Europe than in North America. The most significant change in pathogenesis is inflammation of the central nervous system (CNS) and peripheral nervous system (PNS). Furthermore, clinically, it can represent a variety of neurological manifestations, such as meningitis, encephalitis, radiculopathies, and cranial neuritis. However, dementia-like syndrome is an infrequent manifestation of Lyme disease. Our review article aims to summarize the similarities and differences between dementia-like syndrome in LNB and that in primary neurodegenerative diseases, as well as to look for a correlation between the pathogenesis of the disease and the possibility of developing dementia-like syndrome. The world literature lacks sufficiently convincing data on the relationship between spirochete infection and primary dementia syndromes. However, cases of secondary dementia syndrome due to nervous system involvement as well as post-treatment have been described. A thorough examination, medical history, laboratory and imaging studies, cerebrospinal fluid (CSF) examination, MRI, and fludeoxyglucose-18-positron emission tomography (FGD-PET) are required to differentiate between these syndromes.

## Introduction and background

Lyme disease (LD) is the leading tick-borne illness in the Northern Hemisphere. Its causative agent is a spirochete of the genus *Borrelia *(*B. burgdorferi (Bb)*, *B. garinii*, and *B. afzelii*) [1]. The vector of transmission is the tick *Ixodes *spp. [1]. The LD bacterium, named after its discoverer Wilhelm Burgdorfer in 1982, was first reported in Lyme, Connecticut, USA [2, 3].

Lyme disease is a multisystem disorder demonstrating fever, headache, joint pain, muscle weakness, fatigue, and a typical skin rash. When the skin is involved, it can represent three forms: erythema migrans (EM), borrelial lymphocytoma (BL), and acrodermatitis chronica atrophicans (ACA) [4]. Other typical manifestations involve joints (Lyme arthritis), the heart (Lyme carditis), and the neurological system (Lyme neuroborreliosis (LNB)) [5].

In the context of the epidemiological distribution of the *Borrelia *species, a broad difference may explain the discrepancy in the clinical course of LD in distinct parts of the world. To illustrate that, in North America, *B. burgdorferi* is the only pathogen that causes disease in humans, and the infection’s prevailing manifestations are Lyme arthritis and EM. At the same time, it occasionally leads to LNB in association with *B. burgdorferi* [5]. On the other hand, in Europe, five species have been shown to cause human disease: *B. garinii*, *B. afzelii*, *B. burgdorferi sensu stricto*, *B. spielmanii*, and *B. bavariensis*. *Borrelia afzelii* is more commonly associated with EM, and *B. garinii* and *B. bavariensis* occur more frequently in LNB [6, 7]. The neurotropism of *Borrelia *is, therefore, specific to particular species, and disparities between representatives of the species exist [8].

The aim of our narrative review is twofold: firstly, to present the known pathogenetic mechanisms of LNB and their possible relationship to the development of dementia syndrome in LD; secondly, to compare the different types of primary dementia syndromes with those of LNB.

## Review

Pathogenesis of LNB

*Borrelia *expresses numerous molecules, such as outer surface protein A (OspA) and OspC, that are necessary for its development in ticks [9]. Both molecules are highly immunogenic, and after entering the blood circulation of the mammal host, *Borrelia* "hides'' them to avoid the immune response [9,10]. Further, it has been found that only OspA-negative *Borrelia* can infect mammals [11]. OspC is initially required to bind to the complement inhibitory protein Salp15 from the tick's saliva [12, 13]. In addition, the pathogen possesses a wide variety of mechanisms that enable evasion of the host’s immune system. Additionally, the complement-blocking proteins that the bacteria produce include the host complement regulatory protein CD59 (protectin), the factor H-binding outer surface protein E paralogs [14], and the complement regulatory-acquiring surface proteins (CRASPs) [9,15,16]. These particles increase interleukin (IL)-10 secretion in mononuclear cells [17]. Likewise, *Borrelia* can block opsonization by synthesizing soluble antigens that bind to antibodies [18]. Similarly, bacteria can bind to various proteins, such as decorin and fibronectin, which allow them to hide in immune-privileged tissues like the extracellular matrix [9, 19]

The entry of *Borrelia *into the central nervous system (CNS) needs to be better studied, although two main pathways have been suggested: circulatory and peripheral nerves [9]. Once in the CNS, *Borrelia *encounters monocytes, macrophages, and dendritic cells that synthesize proinflammatory cytokines (IL-6, IL-8, IL-12, IL-18, or interferon (IFN)-γ) and chemokines (CCL19, CCL21, CXCL12, and CXCL13) [20]. Chemokines attract B-cells and lead to B-cell infiltration [21]. In addition to B-cells, activated CD8+ and CD4+ T cells can also be detected [22]. Thus, it leads to lymphocytic pleocytosis, which can be observed in the cerebrospinal fluid (CSF) [21]. Non-human primates (NHPs) have been used in experimental studies to recreate aspects of the pathogenesis of LNB. According to observations supported by positive CSF cultures of *B. burgdorferi* and pleocytosis, LNB is linked to direct spirochetal invasion [23,24]. Another way in which *B. burgdorferi* infection can affect the nervous system is through molecular mimicry. This occurs when antibodies to *B. burgdorferi flagellin* are produced in the patient's body and then react with neural antigens [25]. Recent studies have explored the role of the B-cell-attracting chemokine CXCL13 as a diagnostic marker in patients with early LNB. It was elevated in nearly all of these patients before detecting anti-*Borrelia *antibodies. Furthermore, CXCL13 levels tend to decrease rapidly after the start of antibiotic therapy, which is not the case regarding the anti-*Borrelia *antibodies [26]. CXCL13 can also be detected in other manifestations of LD, such as borrelial lymphocytoma [27]. In some other neurologic manifestations of LD, the CXCL13 assay is negative, for example, in patients with Lyme encephalopathy [28]. It is, however, important to note that elevated CXCL13 in the CSF can also be a marker for noninfectious neurologic diseases such as aseptic meningitis [26].

Parthasarathy et al. (2023) [29] examined the significance of the fibroblast growth factor (FGF)/FGF receptor (FGFR) system in *Borrelia*-induced microglial neuroinflammation. It was found that the pathogen played an essential role in the pathogenesis of primary dementia syndromes and their complications. In some cases, this system may promote a pro-inflammatory response and, in others, an anti-inflammatory one. If this system excessively promotes pro-inflammatory responses, it could contribute to neurodegenerative disorders such as Alzheimer’s disease. Understanding its role may have therapeutic use in developing drugs that target it. FGFR1 also contributes to inflammation mediated by non-viable *B. burgdorferi*[29]. 

Stages and manifestation

Lyme disease is generally classified as an early localized infection, an early disseminated infection, or a late disseminated infection [30]. Joint pain and fever are common symptoms in the first stage, which develop days to weeks after the initial tick bite [24]. In the phase of an early disseminated infection, 10%-15% of untreated patients can develop neurological disorders referred to as early LNB [31]. Such manifestations appear four to six weeks postexposure and present as radiculopathy [31], lymphocytic meningitis, mononeuritis multiplex, and otolaryngological symptoms (tinnitus, vertigo, and hearing loss) [1]. Lyme meningoradiculitis (Bannwarth's syndrome), with intense pain, frequent paresis, and headaches, is the most widespread form of the disease [32]. In the USA, the most common symptom of early disseminated neuroborreliosis is facial palsy, in association with pleocytosis and other CSF abnormalities [33]. Lyme meningitis in children is also relatively frequent [34].

In the late disseminated infection stage, neurological manifestations are associated with Lyme encephalopathy [35], a syndrome involving cognitive difficulties as well as memory loss and personality changes, which is a topic of controversy in the USA [5]. In Europe, cases of late disseminated LNB have been reported with both peripheral manifestations (mononeuropathy, radiculopathy) and central nervous system involvement (cerebral vasculitis, encephalomyelitis with spastic syndrome) [36]. The cases of predominantly sensory peripheral neuropathy with paresthesia in Europe are associated with ACA [37]. Disseminated encephalomyelitis most often manifests itself with spastic syndrome and symptoms of fatigue and gait disturbances similar to those in multiple sclerosis [37]. Dementia-like syndromes and cognitive disturbances in the USA have been observed in the context of the aforementioned encephalopathy and as a post-treatment LD syndrome [38]. In Europe, they are regarded as a rare complication of cerebral vasculitis-like involvement and also in the context of post-treatment Lyme disease syndrome (PTLDS) [37,39].

Lyme neuroborreliosis-associated dementia-like syndrome

Dementia syndrome in general is an acquired and progressive loss of cognition in multiple cognitive domains sufficiently severe to affect social or occupational function [40]. Dementia is an umbrella syndrome associated with memory impairment and loss, cognitive disturbances, and personality changes to such an extent that they interfere with daily life. Symptoms can be related to Alzheimer’s disease, frontotemporal dementia (FTD), vascular dementia (VD), Lewy body dementia (LDB), Huntington’s disease, mixed dementia syndromes, and others. The most common clinical form of the disease is “mixed dementia, usually a combination of a common age-related neurodegenerative disease, most often Alzheimer’s disease and VD [40]. On the other hand, dementia-like syndrome can be an extremely rare manifestation of LNB, and the current understanding of its pathogenesis, manifestations, and treatment is limited. Some studies have concluded that LNB patients did not have increased long-term risks of dementia, Alzheimer’s disease, Parkinson’s disease, motor neuron diseases, epilepsy, or Guillain-Barré, but might have an increased short-term risk of epilepsy and Guillain-Barré syndrome [41]. 

Nevertheless, dementia-like syndromes have been occasionally reported in the last few years, and our article aims to summarize the similarities as well as differences between dementia associated with LNB and primary neurodegenerative dementia syndromes such as those mentioned above.

In order to associate existing dementia with a previous *B. burgdorgeri* infection, clinicians must execute a serology evaluation test and also immunology for intrathecal antibody production. A positive intrathecal anti-*Borrelia* antibody index (AI) is necessary to support the diagnosis. Since LNB is an inflammatory disease of the CNS, it is characterized by the phenomenon of lymphocytic pleocytosis in the CSF, an increased number of leukocytes at the expense of lymphocytes and plasma cells, along with an increased protein level (Table 1) [42].

**Table 1:** Diagnosis of Lyme neuroborreliosis (LNB) based on the European Federation of Neurological Societies (EFNS) guidelines For a diagnosis of definite Lyme neuroborreliosis, all three criteria must be met; for a diagnosis of possible Lyme neuroborreliosis, at least two of the three criteria must be met.

Criteria	Definite neuroborreliosis	Possible neuroborreliosis		
Intrathecal antibody production	+	+	+	-
Cerebrospinal fluid pleocytosis	+	+	-	+
Neurological symptoms suggestive of LNB without other obvious reasons	+	-	+	+

A study in Strasbourg involved 20 patients exhibiting dementia syndrome and a positive AI. A ceftriaxone dosage of 2 g/daily was administered to all of them, and they were divided into two groups. Group 1 (N = 7) exhibited a stable or improved course of their dementia syndrome. Group 2 (N = 13) demonstrated a deterioration during treatment, and all participants from this group showed evidence of an underlying primary dementia syndrome such as Alzheimer’s disease, Lewy body disease, frontotemporal dementia, and vascular dementia. They concluded that both pure Lyme dementia and primary neurodegenerative dementia associated with positive AI exist; fortunately, Lyme dementia without other neurocognitive comorbidities has a better outcome after the antibiotic treatment [39].

Post-treatment Lyme disease syndrome

In recent years, the subject matter of PTLDS has been excessively studied, and literature reports have significantly increased [1,38,43]. Post-treatment Lyme disease syndrome refers to a long-term condition where individuals who have been successfully treated for Lyme disease continue to experience memory loss, language difficulties, and impaired mental processing [44]. It is believed that both the bacterium itself and the persistence of bacterial debris trigger an immune response to the infection [28]. Although there is general consensus that PTLDS is a syndrome following treated LNB, there is still controversy surrounding its treatment with long-term antibiotic therapy [45]. The evidence provided in the medical literature so far is unanimous in confirming that long-lasting antibacterial therapy does not benefit patients with PTLDS [28].

Lyme neuroborreliosis and other types of dementia

Alzheimer’s disease is the main cause of dementia and one of the great healthcare challenges of the 21^st^ century. It presents with typical (memory impairment and executive dysfunction interfering with daily-life activities) and atypical manifestations (language, visual, practical, or executive problems before and more pronounced than memory deficits) [46]. In a case report from 2023, a 75-year-old patient was first admitted to an Alzheimer’s care unit due to psychiatric disturbances and aggressive behavior. The patient underwent multiple different examinations and diagnostic tests where meningitis, limbic encephalitis, and syphilis were excluded (Creutzfeldt-Jakob disease was also a hypothesis). The patient scored 22/30, 3/5, 35/100, and 7/28 on the Mini-Mental State Exam (MMSE), Clinical Dementia Rating (CDR®), Barthel index, and Tinetti scale, respectively. The positive serology tests for *Borrelia *confirmed the diagnosis, as the patient also had arthritis and reported a tick bite six months prior (EM was not present). After a complete 21-day course of antibiotic treatment with intravenous ceftriaxone (2 g/daily), followed by oral doxycycline (200 mg/daily for seven days), his neurological symptoms improved, and his newly assessed scores were 29/30, 2/5, 62/100, and 25/28 [47].

Kristoferitsch et al. [37] present a series of 10 clinical cases where the onset of dementia-like syndrome manifests itself faster compared to primary dementia syndrome. Patient number 10, a 76-year-old female, was admitted with symptoms of progressing cognitive decline, weight loss, gait disturbance, and tremors over the last 12 months. Since the patient also suffered from mild hypertension, vascular encephalopathy was thought to be the most likely cause of the decline, but this was excluded. Her significant weight loss (20 kg/year) was more indicative of LD in contrast to Alzheimer’s disease (mean weight loss 0.9 kg/year). She was diagnosed with LNB when further CSF examinations disclosed a highly elevated AI. The patient was treated with 2 g of ceftriaxone daily for three weeks, and the symptoms rapidly improved. 

Frontotemporal dementia is common in patients younger than 65 years. It is characterized by frontotemporal atrophy on diagnostic imaging and is classified into the following subtypes: behavioral-variant frontotemporal dementia, nonfluent variant primary progressive aphasia, and semantic-variant primary progressive aphasia [48]. Depending on the type of FTD, we have different types of symptoms, consisting of behavioral change, including inappropriate ones, apathy, aphasia, language deficit, etc. [49]. Batistta et al. [50] describe a case of a 61-year-old patient diagnosed with borreliosis at the age of 57 years, treated with doxycycline 200 mg/day for 21 days. After a few weeks, depressive episodes and apathy appeared, so she started treatment at another center with doxycycline 200 mg/day for 14 days. After the course of treatment, anxiety and panic attacks appeared, and depressive episodes worsened. Treatment with benzodiazepine and antidepressant therapy was started; it did not affect the symptoms; socially inappropriate behavior, excessive smoking, and food intake appeared. Cerebrospinal fluid tests were performed, of which IgM and IgG were negative; the polymerase chain reaction (PCR) test was negative for the presence of *B. burgdorferi*. Both MRI and fludeoxyglucose-18-positron emission tomography (FGD-PET) were performed with signs characteristic of FTD. 

Dementia with Lewy bodies is the third most common dementia after Alzheimer's disease and VD. It has an earlier onset than Alzheimer's dementia and is often associated with visual hallucinations, attention deficit, rapid progression, and extrapyramidal symptomatology [51]. Gadilka et al. [52] present a case of a 69-year-old female patient who was diagnosed with LD at the age of 54 and treated for 10 days with doxycycline 200 mg/day until her condition improved. After four years, cognitive changes as well as photophobia and paresthesias appeared. Sputum tests were performed, which showed no pleocytosis and no intrathecal production of antibodies to *Borrelia*. Given the early motor disturbances, rapid eye movement (REM), behavioral disorder, paranoia, and personality changes, the diagnosis of LDB was made, but no readily available convincing evidence of a link between LNB and LDB is presented [53].

Another common type of dementia is VD (about 15% of cases), which has similar symptoms to Alzheimer's dementia and has several subtypes: multi-infarct, strategic infarct, hemorrhagic, hereditary, hypoperfusion, and small vessel dementia [54]. One of the risk factors for developing VD is a history of stroke, with 15%-30% of the people surviving stroke developing dementia over time [55]. No cases of VD resulting from LNB have been described. However, cases of stroke resulting from acute LNB have been reported, with a putative mechanism being the development of small vessel vasculitis due to perivascular and vascular infiltration with lymphocytes (Table 2) [56].

**Table 2:** Clinical characteristics of different types of dementias

Dementia type	Medical history	Main clinical features	Neurological status	Diagnostic imaging	Cerebrospinal fluid (CSF) findings
Alzheimer’s disease (AD)	Gradual, insidious onset	Memory and cognition impairment, loss of independence in daily activities	Initially normal	Global atrophy (especially the hippocampus and parahippocampal gyrus)	Elevated tau, p-tau, and beta-amyloid 42 (Aß42)
Frontotemporal dementia	Behavioral, emotional, and personality changes, aphasia	Expressive aphasia, apathy, euphoria, depression, disinhibition	Vertical gaze palsy, axial rigidity, dystonia, alien hand	Atrophy of frontal, insular, and/or temporal lobes; spares posterior lobe	Higher levels of Aß42, and lower t-tau/Aß42 and p-tau/Aß42 values compared with AD
Vascular dementia	History of transient ischemic attack/stroke, vascular risk factors, hemiparesis, urinary incontinence	Cognitive slowing could spare memory, apathy, or depression	Motor slowing, spasticity	Lacunar/non-lacunar infarction, white matter lesions	Elevated interleukin-6 and C-reactive protein, elevated levels of lipocalin 2 (LCN2) compared to other dementias
Lewy body dementia	Visual hallucinations, rapid eye movement (REM), sleep disorders, parkinsonism	Extrapyramidal signs such as Parkinsonism which includes rigidity, tremors, gait disturbance, autonomous nervous system symptoms	Parkinsonism	Posterior parietal atrophy occipital hypometabolism on fluorodeoxyglucose-positron emission tomography (FDG-PET), hippocampi larger than in AD	Elevated levels of Lewy bodies (α-synuclein), tau protein, and other proteins (ubiquitin, neurofilament protein), amyloid deposits
Lyme neuroborreliosis-associated dementia	Tick bite history, fever, joint pain, erythema migrans	Radiculopathy, lymphocytic meningitis, mononeuritis, multiplex otolaryngological symptoms (tinnitus, vertigo)	Bannwarth’s syndrome, sensory peripheral neuropathy with paresthesia associated with acrodermatitis chronica atrophicans Lyme encephalitis	An MRI can show areas of inflammation, with increased signal on T2 and fluid-attenuated inversion recovery (FLAIR) imaging	Lymphocytosis, monocytosis, or both intrathecal production of anti-*Borrelia *antibodies

We have only two large population-based studies, one in the USA, which found no epidemiological association between the occurrence of Alzheimer's disease and LNB [57] (however, LNB is more common in the European population), and another in Denmark, which found no risk of developing dementia syndrome in patients with LNB [41]. However, the risk was assessed for a period of one year, with patients on treatment during this time.

A differential diagnosis of the various forms of dementias, including Lewy body disease, is a challenging and multi-faceted process. It requires consideration of the patient's overall physical condition and the findings of a comprehensive neurological examination, as well as the results of advanced diagnostic techniques. Investigations may include imaging (e.g., atrophy of the cerebral cortex or other parts of the brain, or traces of inflammation) or a CSF examination (to look for characteristic proteins, such as tau, beta-amyloid 42, IL-6, α-synuclein, and Ab).

## Conclusions

Current knowledge of the dementia-like syndrome caused by LNB is still quite limited. Only a few cases have been described in the medical literature, and not many dedicated studies have been conducted on this subject. We suggest that this may be due to several challenging factors: the frequent confusion between LNB and other neurological diseases, the substantial difficulty in the differential diagnosis of various dementia syndromes, and the selection of appropriate patients/cohorts of patients, as dementia syndrome usually develops slowly, suggesting the involvement of patients who may have already been in the initial stages of primary dementia syndrome. Therefore, the topic of dementia syndrome and LNB is a challenging one, and more control studies are needed. Attention in the diagnosis of a secondary dementia-like syndrome associated with LNB should be directed to generalized somatic manifestations (fever) that are usually absent in primary dementia syndromes, possibly a CSF examination for evidence of intrathecal antibody production, especially if the patient has previously visited a region endemic for LD. Proper differentiation with other neurological or infectious diseases is essential to providing our patients with the best possible care.
